# Glycoform-Selective Prion Formation in Sporadic and Familial Forms of Prion Disease

**DOI:** 10.1371/journal.pone.0058786

**Published:** 2013-03-19

**Authors:** Xiangzhu Xiao, Jue Yuan, Stéphane Haïk, Ignazio Cali, Yian Zhan, Mohammed Moudjou, Baiya Li, Jean-Louis Laplanche, Hubert Laude, Jan Langeveld, Pierluigi Gambetti, Tetsuyuki Kitamoto, Qingzhong Kong, Jean-Philippe Brandel, Brian A. Cobb, Robert B. Petersen, Wen-Quan Zou

**Affiliations:** 1 Department of Pathology, Case Western Reserve University School of Medicine, Cleveland, Ohio, United States of America; 2 Université Pierre et Marie Curie-Paris 6, Centre de Recherche de l’Institut du Cerveau et de la Moelle épinière (CRICM), UMRS 975, Equipe Maladies à Prions – Maladie d’Alzheimer; Inserm, U 975; CNRS, UMR 7225; and AP-HP, Hôpital de la Salpêtrière, Cellule Nationale de Référence des maladies de Creutzfeldt-Jakob, Paris, France; 3 Virologie Immunologie Moléculaires, UR892, INRA, Jouy-en-Josas, France; 4 UF de Génétique Moléculaire, pôle B2P, Hôpital Lariboisière, Paris, France; 5 Central Veterinary Institute of Wageningen UR, Lelystad, the Netherlands; 6 National Prion Disease Pathology Surveillance Center, Case Western Reserve University School of Medicine, Cleveland, Ohio, United States of America; 7 Division of CJD Science and Technology, Department of Prion Research, Center for Translational and Advanced Animal Research on Human Diseases, Tohoku University Graduate School of Medicine, Sendai, Japan; 8 Department of Neurology, Case Western Reserve University School of Medicine, Cleveland, Ohio, United States of America; 9 Department of Neuroscience, Case Western Reserve University School of Medicine, Cleveland, Ohio, United States of America; 10 Department of Critical Care Medicine, the First Affiliated Hospital, Nanchang University, Nanchang, Jiangxi Province, The People’s Republic of China; 11 National Center for Regenerative Medicine, Case Western Reserve University School of Medicine, Cleveland, Ohio, United States of America; 12 State Key Laboratory for Infectious Disease Prevention and Control, National Institute for Viral Disease Control and Prevention, Chinese Center for Disease Control and Prevention, Beijing, People’s Republic of China; National Research Council of Italy, Italy

## Abstract

The four glycoforms of the cellular prion protein (PrP^C^) variably glycosylated at the two N-linked glycosylation sites are converted into their pathological forms (PrP^Sc^) in most cases of sporadic prion diseases. However, a prominent molecular characteristic of PrP^Sc^ in the recently identified variably protease-sensitive prionopathy (VPSPr) is the absence of a diglycosylated form, also notable in familial Creutzfeldt-Jakob disease (fCJD), which is linked to mutations in PrP either from Val to Ile at residue 180 (fCJD^V180I^) or from Thr to Ala at residue 183 (fCJD^T183A^). Here we report that fCJD^V180I^, but not fCJD^T183A^, exhibits a proteinase K (PK)-resistant PrP (PrP^res^) that is markedly similar to that observed in VPSPr, which exhibits a five-step ladder-like electrophoretic profile, a molecular hallmark of VPSPr. Remarkably, the absence of the diglycosylated PrP^res^ species in both fCJD^V180I^ and VPSPr is likewise attributable to the absence of PrP^res^ glycosylated at the first N-linked glycosylation site at residue 181, as in fCJD^T183A^. In contrast to fCJD^T183A^, both VPSPr and fCJD^V180I^ exhibit glycosylation at residue 181 on di- and monoglycosylated (mono181) PrP prior to PK-treatment. Furthermore, PrP^V180I^ with a typical glycoform profile from cultured cells generates detectable PrP^res^ that also contains the diglycosylated PrP in addition to mono- and unglycosylated forms upon PK-treatment. Taken together, our current *in vivo* and *in vitro* studies indicate that sporadic VPSPr and familial CJD^V180I^ share a unique glycoform-selective prion formation pathway in which the conversion of diglycosylated and mono181 PrP^C^ to PrP^Sc^ is inhibited, probably by a dominant-negative effect, or by other co-factors.

## Introduction

Prion diseases are a group of fatal transmissible spongiform encephalopathies affecting both animals and humans. Human prion diseases are highly heterogeneous: They can be inherited, sporadic, or acquired, and include various forms of Creutzfeldt-Jakob disease (CJD), Gerstmann-Sträussler-Scheinker (GSS) disease, fatal insomnia, and kuru. Moreover, regardless of differences in phenotypes, they are all caused by infectious pathologic prions (PrP^Sc^) that are derived from the cellular prion protein (PrP^C^) through a conformational transition [1]. The partially proteinase K (PK)-resistant PrP27–30 (PrP^res^) is the molecular hallmark of all human prion diseases. On Western blots three major PrP bands are typically observed but composed of a di-, two mono-, and an unglycosylated PrP glycoforms because the two individually monoglycosylated PrP at asparagine residues N181 and N197 partially overlap. There are a few exceptions [2]–[5]. Of them, all but one are associated generally with PrP mutations showing different PrP banding patterns, as in GSS and familial CJD. GSS is characterized by the presence of additional small PK-resistant PrP fragments, whereas fCJD linked to either PrP^T183A^ or PrP^V180I^ mutations exhibits a PrP^res^ that lacks the diglycosylated PrP species [2]–[4].

The abnormal PrP associated with the recently identified prion disease termed variably protease-sensitive prionopathy (VPSPr) has highly distinctive features [6], [7], including a one that was initially reported as an atypical sCJD by Giaccone et al [8]. Although there is no PrP mutation in the open reading frame of the PrP gene, VPSPr is associated with a PrP^res^ that bears three of the characteristics of inherited rather than sporadic prion diseases. First, the diglycosylated PrP^Sc^ in VPSPr is virtually undetectable, as it is also with PrP^res^ in fCJD^V180I^ and fCJD^T183A^ [3], [4], [7]. Second, VPSPr is characterized by the presence in the brain of more than three PrP^res^ fragments including a ∼7 kDa fragment, a characteristic of GSS [2], [7]. However, in marked contrast to PrP^res^ in GSS, PrP^res^ in VPSPr is preferentially detected with the 1E4 antibody instead of the widely used 3F4 antibody, forming a pathognomonic five-step ladder-like PrP electrophoretic profile [7]. Finally, in some VPSPr cases, a positive family history of cognitive impairment was observed [6], [7]. Clearly, the PrP^Sc^ associated with VPSPr is distinct from the prion strains associated with other sporadic prion diseases. The molecular mechanism underlying the formation of the peculiar prion in VPSPr has yet to be determined.

Compared to PrP in the most common sporadic CJD (sCJD), a significant decrease in the ratio of diglycosylated PrP to monoglycosylated PrP treated with or without PK was reported in fCJD^T183A^ previously [3]. This is because the T183A PrP mutation completely abolishes the first N-linked glycosylation site at residue 181 (N181) [9]–[11] and the detected diglycosylated PrP is derived only from wild-type PrP (3, the current study). In contrast, the PrP glycoforms in VPSPr appear typical prior to PK-treatment; however, there is no detectable diglycosylated PrP^Sc^ after PK-treatment. As with VPSPr, the molecular mechanism underlying the absence of the diglycosylated PrP in fCJD^V180I^ is unclear [4]. Using a combination of *in vivo* and *in vitro* assays, our current study indicates that the absence of the diglycosylated PrP^Sc^ in both VPSPr and fCJD^V180I^ results from a glycoform-selective prion formation pathway associated with the inability of the di- and mono-glycosylated PrP^C^ at N181 to convert into PrP^Sc^ in the brain.

## Results

### Both inherited CJD^V180I^ and sporadic VPSPr exhibit no diglycosylated PrP^res^

**Figure 1:**
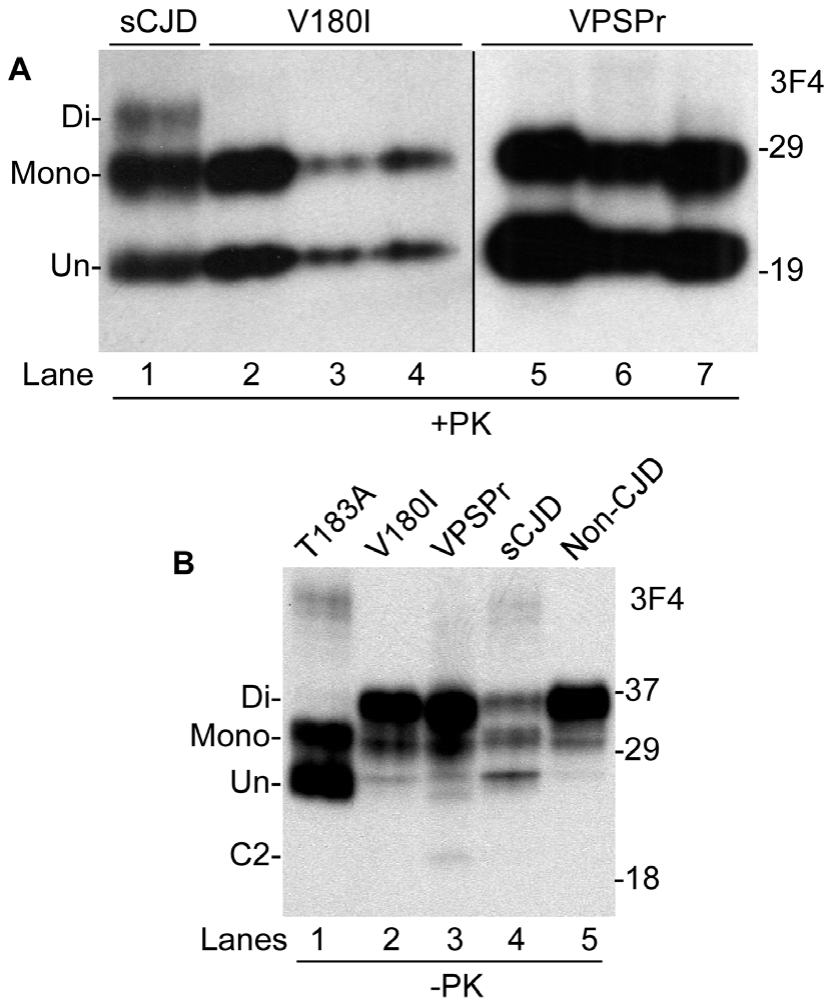
Detection of PK-treated and untreated PrP with 3F4. (*A*) Brain homogenates from three fCJD^V180I^ (one 129MM and two 129MV, lanes 2–4) and three VPSPr-129MM cases (lanes 5–7) were treated with PK at 10 µg/ml prior to Western blotting with 3F4. A sCJDMM2 case was used as a control (lane 1). (*B*) PrP in brain homogenates without PK-treatment from fCJD^T183A^, fCJD^V180I^, VPSPr, sCJD and non-CJD was examined by Western blotting.

In contrast to sCJD, both fCJD^V180I^ and VPSPr exhibit mono- and un-glycosylated PK-resistant PrP bands but virtually no diglycosylated PrP when probed with the 3F4 antibody (Fig. 1A). However, in the samples that were not treated with PK (Fig. 1B), diglycosylated PrP was readily detectable not only in sCJD and non-CJD but also in fCJD^V180I^ and VPSPr. The fCJD^T183A^ exhibited a very faint diglycosylated PrP band that was visible in over-exposed blots and is from the wild-type allele as reported previously [3].

### Lack of diglycosylated PrP^res^ is attributable to loss of glycosylation at the first N-linked glycosylation site in fCJD^V180I^ and VPSPr

**Figure 2:**
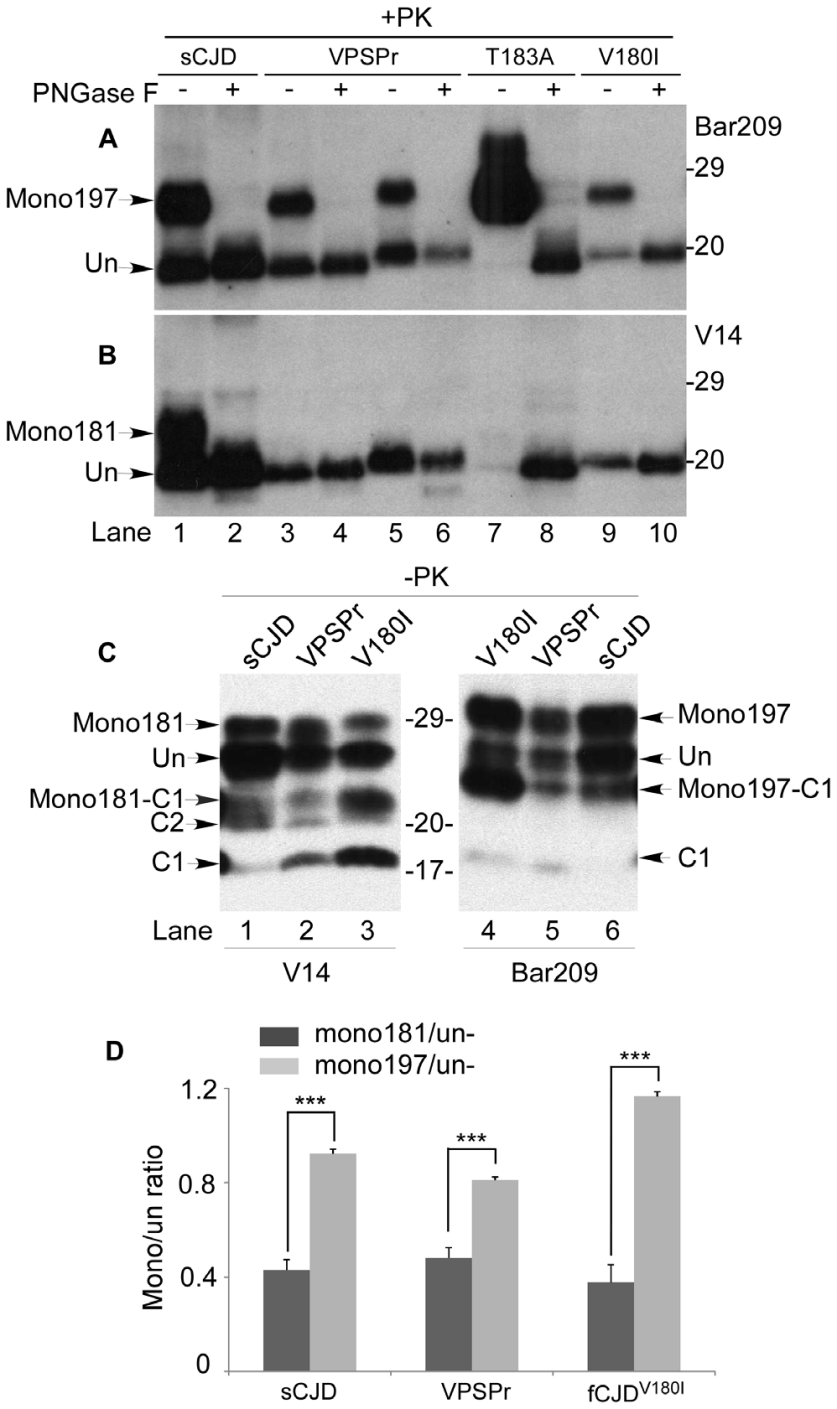
Detection of two individual monoglycosylated PrP either at N181 or N197. (*A*) and (*B*) sCJDMM2 (lanes 1, 2), two VPSPr (129MM: lanes 3, 4; 129MV: lanes 5, 6), fCJD^T183A^ (lanes 7, 8) and fCJD^V180I^ (lanes 9, 10) treated with PK or PK plus PNGase F were probed with Bar209 (*A*) or V14 (*B*). (*C*) PrP from VPSPr, fCJD^V180I^, and sCJD was examined with V14 (lanes 1–3) or Bar209 (lanes 4–6). (*D*) Ratio of mono- (either mono181 or mono197) to unglycosylated PrP by densitometric analysis based on three independent experiments, one of which is presented in (*C*). The black bar represents mono181:unglyc PrP, while the grey bar represents mono197:unglyc PrP from sCJD, fCJD^V180I^ or VPSPr. *** *p* <0.005.

To investigate whether and how the two individual N181 and N197 sites are associated with the lack of the diglycosylated PrP^res^ in fCJD^V180I^ and VPSPr, we probed PrP treated with PK or PK plus PNGase F using V14 and Bar209 antibodies that have been demonstrated to distinguish mono181 and mono197 but not di- and unglycosylated PrP (Fig. S1 and S2) [11]–[14]. On the blot probed with the Bar209 antibody that specifically detects mono197, both mono197 and unglycosylated PrP migrating at ∼26–28 kDa and ∼19–20 kDa, respectively, were detected in all samples treated with PK alone except for fCJD^T183A^ that had a significantly under-represented unglycosylated PrP (Fig. 2A). fCJD^T183A^ exhibited a dominant PrP band migrating at ∼26–28 kDa, corresponding to mono197. In the samples treated with PK plus PNGase F, only one PrP band migrating at ∼19–20 kDa was observed, corresponding to un- and deglycosylated PrP species (Fig. 2A). Therefore, mono197 is converted into PrP^res^ in the four prion diseases examined.

On the blot probed with the V14 antibody that specifically detects mono181 [11], [12], the PrP band migrating at ∼24–26 kDa corresponding to mono181 was detected in sCJD in addition to the band migrating at ∼19–20 kDa corresponding to the unglycosylated PrP (Fig. 2B). In contrast, as in fCJD^T183A^, no mono181 was detected in both fCJD^V180I^ and VPSPr, while the unglycosylated PrP migrating at ∼19–20 kDa was detected in all diseases (Fig. 2B). Only the faint under-represented unglycosylated PrP band migrating at ∼19–20 kDa was detected in fCJD^T183A^. After treatment with PK plus PNGase F, the un-/de-glycosylated PrP band migrating at ∼19–20 kDa was detected in all cases examined (Fig. 2B). These results suggest that the lack of diglycosylated PrP^res^ in both VPSPr and fCJD^V180I^ is associated with the absence of the PK-resistant mono181 as found in fCJD^T183A^. Notably, the migration of PrP^res^ from VPSPr-129MV and fCJD^V180I^ was slightly slower than that of PrP^res^ from sCJD type 2, VPSPr-129MM and fCJD^T183A^ when probed with V14 and Bar209 [Fig. 2A and 2B], although it was very similar when probed with 3F4 (Fig. 1A). What is responsible for the discrepancy in the migration of the core PrP^res^ observed when probing with these antibodies remains to be determined in the future.

To investigate whether there are any changes in the two monoglycosylated PrP species in the samples prior to PK-treatment, we examined PrP profiles from VPSPr and fCJD^V180I^ with the two antibodies in untreated samples. In contrast with PK-treated PrP, mono181 or mono197 was detected in both VPSPr and fCJD^V180I^ in addition to C2 and C1, two fragments identified previously in human brains [11], [15], similar to those detected in the sCJD control (Fig. 2C). Thus, in contrast to fCJD^T183A^, both VPSPr and fCJD^V180I^ were found to contain mono181 before PK-treatment. In combination these data suggests that mono181 is not converted into PrP^res^ in the two diseases and that mono181 of VPSPr and fCJD^V180I^ is different from that of sCJD. Notably, mono197 from all conditions migrated more slowly than did mono181 at ∼0.5–1 kDa, consistent with previous observations [11], [12]. Moreover, the ratio of mono197 intensity to unglycosylated PrP was significantly greater than that of mono181 to unglycosylated PrP in all three diseases (sCJD: 0.92 ± 0.02 vs. 0.43 ± 0.08, *p* < 0.001; VPSPr: 0.81 ± 0. 02 vs. 0.48 ± 0.04, *p* < 0.001; and fCJD^V180I^: 1.17 ± 0.01 vs. 0.38 ± 0.04, *p* < 0.001) (Fig. 2C and 2D). Therefore, the lack of diglycosylated PrP^res^ in fCJD^V180I^ and VPSPr is directly attributable to the absence of PK-resistant mono181, whereas prior to PK-treatment the proportions of diglycosylated PrP and mono181 do not appear unusual in the two diseases.

### Neither PK-resistant nor PK-sensitive PrP^Sc^ glycosylated at N181 is detected in both VPSPr and fCJD^V180I^

**Figure 3:**
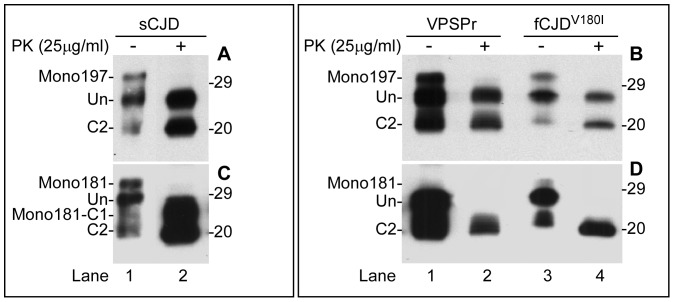
Detection of PrP^Sc^ before and after PK-treatment. PrP in P2 fractions from sCJD, VPSPr and fCJD^V180I^ was subjected to g5p-capture and treatment with or without PK prior to Western blotting with Bar209 (*A* and *B*) or V14 (*C* and *D*).

To determine whether there is a PK-sensitive mono181 PrP^Sc^ in both VPSPr and fCJD^V180I^, we isolated total PrP^Sc^ using the gene 5 protein (g5p) that has proven to specifically capture not only PK-resistant but also PK-sensitive PrP^Sc^ [6], [16]. The PrP^Sc^ captured by g5p was untreated or treated with PK prior to Western blotting and probing with Bar209 or V14. The Bar209 antibody detected three PrP bands in untreated sCJD sample corresponding to mono197 and unglycosylated full-length PrP (Un-) as well as the endogenously N-terminally truncated PrP fragment called C2, respectively (Fig. 3A). After PK-treatment, PK-resistant mono197 and unglycosylated PrP were observed (Fig. 3A). Similar to sCJD, both VPSPr and fCJD^V180I^ also exhibited three PrP bands prior to PK-treatment (Fig. 3B), while the PK-resistant mono197 and unglycosylated fragments appeared upon PK-treatment when probed with Bar209 (Fig. 3B).

When probed with the V14 antibody, mono181, unglycosylated full-length PrP and C2, respectively, were detected in untreated sCJD samples (Fig. 3C). The PK-resistant mono181 and unglycosylated PrP were observed in PK-treated sCJD samples as well. In contrast, mono181 was not detectable in either untreated or PK-treated samples from VPSPr and fCJD^V180I^ with V14 (Fig. 3D). Therefore, neither PK-resistant nor PK-sensitive PrP^Sc^ glycosylated at residue 181 is detectable in either VPSPr or fCJD^V180I^.

### PrP^res^ from fCJD^V180I^ reveals an electrophoretic profile similar to that of VPSPr

**Figure 4:**
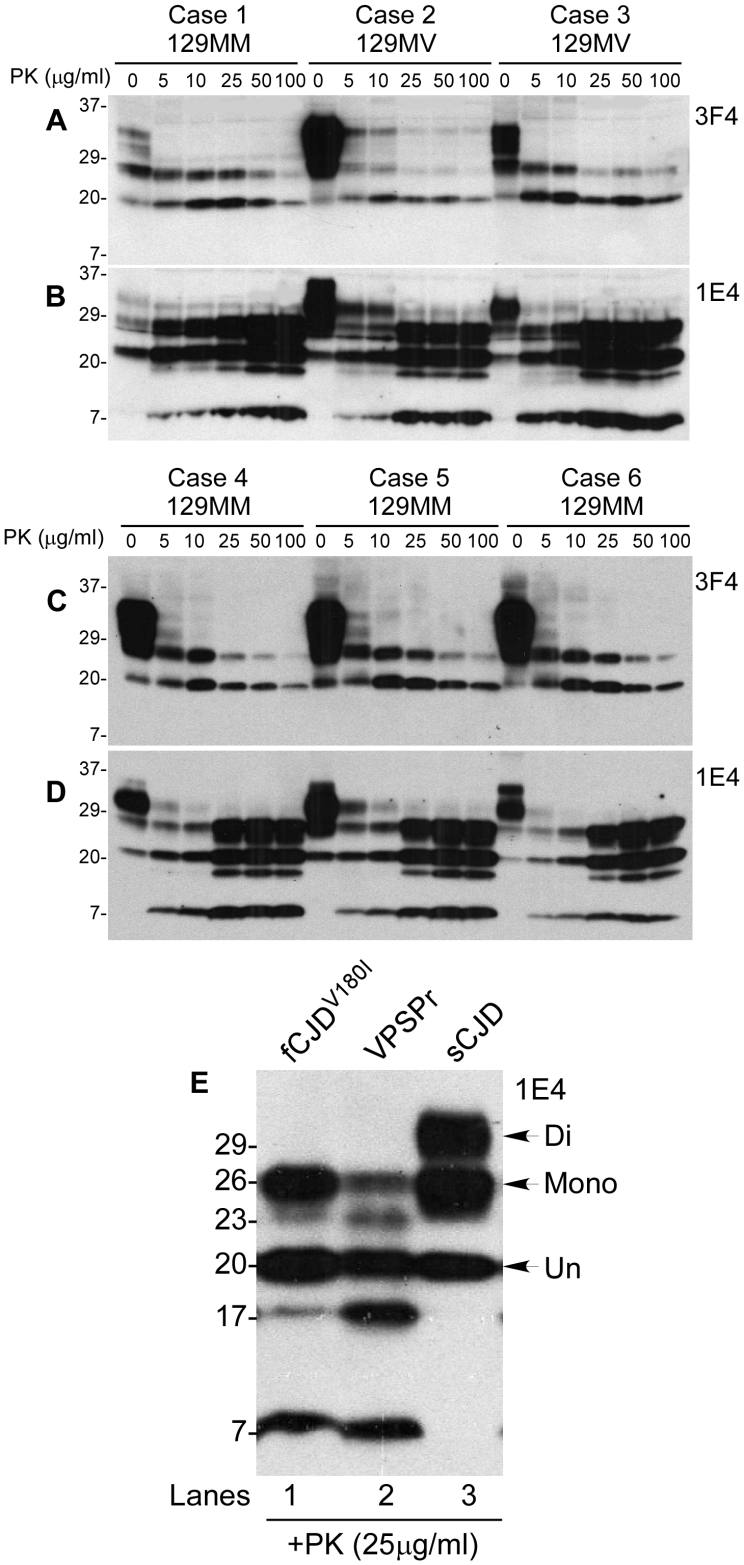
Detection of PrP^res^ in fCJD^V180I^ and VPSPr with 1E4 and 3F4. (*A*–*D*) Brain homogenates from six fCJD^V180I^ cases (four 129MM and two 129MV) were treated with different amounts of PK from 0 to 100 µg/ml prior to Western blotting with 3F4 (*A* and *C*) or 1E4 (*B* and *D*). (*E*) Comparison of PrP^res^ from fCJD^V180I^, VPSPr, and sCJD probing with 1E4.

When probed with 3F4, PrP^Sc^ from all six fCJD^V180I^ cases treated with different amounts of PK exhibited two bands corresponding to mono- and un-glycosylated PrP^res^ and no diglycosylated PrP^res^ (Fig. 4A and 4C), the same as VPSPr-129MM [7]. Moreover, like VPSPr, all cases exhibited the five bands producing a ladder-like electrophoretic profile (LLEP) when probed with 1E4, including a predominant 1E4-preferentially-detectable 7 kDa fragment (Fig. 4B and 4D). PrP^Sc^ from fCJD^V180I^ revealed a higher affinity for 1E4 than for 3F4 (Fig 4A through 4D), another molecular feature of PrP^Sc^ observed in VPSPr. When the PK-treated PrP^Sc^ from the two diseases is compared side by side on the gel, they exhibited virtually identical migration patterns that are different from those of sCJD (Fig. 4E).

### Glycan composition in VPSPr and fCJD^V180I^ is different from that in sCJD

The finding that di- and mono-glycosylated PrP species carrying glycans at N181 are unable to form PrP^Sc^ may suggest that the glycan composition at this site in VPSPr and fCJD^V180I^ is different from that in sCJD. Thus, we compared the binding of *ricinus communis agglutinin* I (RCA-I) to PrP glycans. RCA-I recognizes oligosaccharides ending in galactose/N-acetylgalactosamine (Galβ1-4GlcNAcβ1-R) and has previously been used to compare the differences in glycan composition in different species and prion strains [17]. RCA-I reacted with both di- and mono-glycosylated PrP (Fig. S3A). Compared to that in sCJD, in VPSPr and fCJD^V180I^, the reactivity of RCA-I with monoglycosylated PrP decreased [69.65 (sCJD) vs. 55.82 (VPSPr), *p*  =  0.0051 <0.01; 69.65 (sCJD) vs. 49.49 (fCJD^V180I^), *p*  =  0.0012 <0.005], whereas the reactivity of RCA-I with diglycosylated PrP increased [30.64 (sCJD) vs. 37.70 (VPSPr), *p*  =  0.0012 <0.005; 30.64 (sCJD) vs. 39.44 (fCJD^V180I^), *p*  =  0.00027 <0.001] (Fig. S3B). This result indicates that glycan composition in VPSPr and fCJD^V180I^ is indeed different from that in sCJD by an increased amount of Galβ1-4GlcNAcβ1-R in the diglycosyl moiety.

### There are no detectable differences in profile of glycosylation and truncation between PrP^Wt^ and PrP^V180I^ expressed in cultured cells

**Figure 5:**
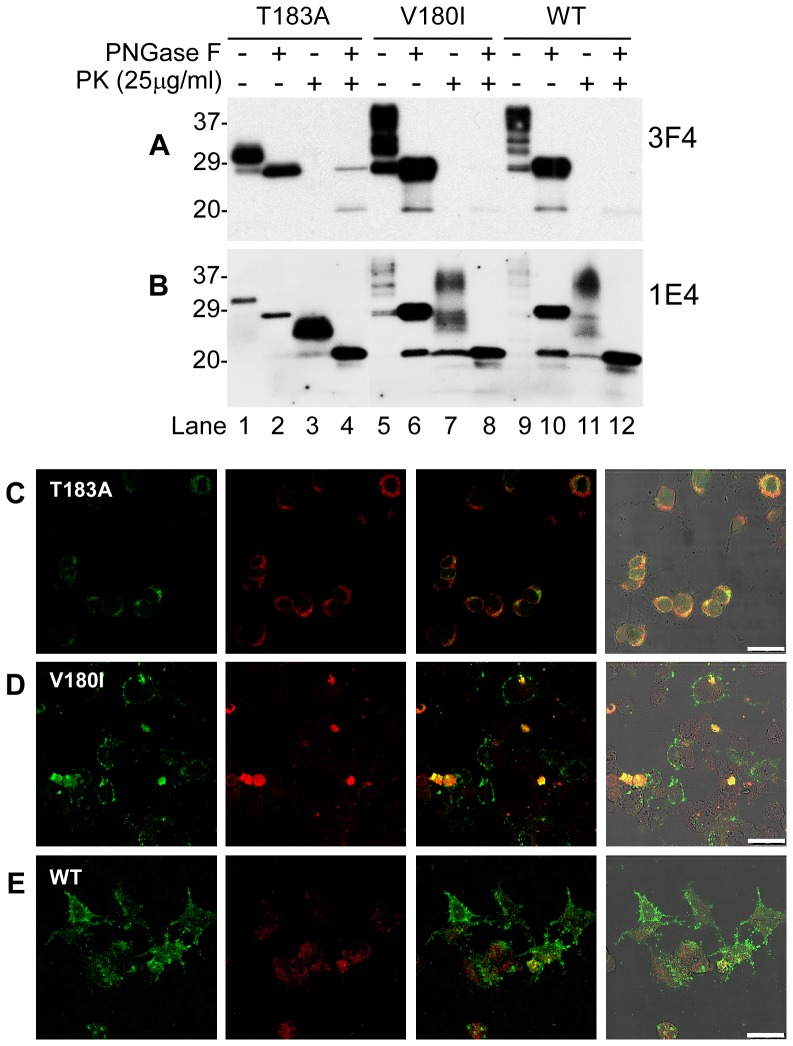
Comparison of glycosylation, proteolysis and distribution of PrP^T183A^, PrP^V180I^, or PrP^Wt^ expressed in cultured cells. Cell lysates from cultured M17 cells expressing PrP^T183A^, PrP^V180I^, or PrP^Wt^ were treated with or without PK and/or PNGase F prior to Western blotting with 3F4 (*A*) or 1E4 (*B*). Subcellular localization of PrP^T183A^, PrP^V180I^ and PrP^Wt^ (*C*, *D*, and *E*). Immunofluorescence staining of cells using 3F4 for PrP (green) and calnexin for ER (red). Virtually all PrP^T183A^ was colocalized with calnexin (*C*). PrP^V180I^ was also colocalized with calnexin, but was found on cell surface equally (*D*). PrP staining was mostly found on the cell surface in cells expressing PrP^Wt^ (*E*). Scale bars: 25 µM.

To determine whether the PrP^V180I^ mutation is directly responsible for the altered glycosylation and proteolytic profiles observed in fCJD^V180I^, human neuroblastoma (M17) cells transfected with human PrP^V180I^, PrP^T183A^, or PrP^Wt^ with valine polymorphism at codon 129 were examined by Western blotting and immunofluorescence confocal microscopy. Western blots showed no significant differences in glycosylation and N-terminal truncation profiles between PrP^Wt^ and PrP^V180I^ before and after treatment with PNGase F when probed with 3F4 antibody (Fig. 5A). As expected [9], [10], PrP^T183A^ exhibited a dominant mono197 band and a minor unglycosylated PrP band. Neither diglycosylated PrP nor mono181 was observed in PrP^T183A^. No PrP^res^ was detected with 3F4 in all three cell lysates treated with PK alone. After treatment with PK plus PNGase F, a faint PK-resistant deglycosylated PrP band migrating at ∼20 kDa was observed in the three lysates, while additional undigested full-length PrP was also detected in PrP^T183A^ (Fig. 5A). In contrast, PrP^res^ was probed in all PrP species treated with PK alone by using 1E4 (Fig. 5B). As reported previously [7], [10], [11], [18], while 1E4 exhibits a poor affinity for untreated PrP, it has a higher affinity for PK-treated PrP compared to 3F4. In PrP^T183A^, a dominant mono197 and a minor unglycosylated PrP but no diglycosylated PrP^res^ were detected, consistent with our previous observations [10], [11]. In contrast with PrP^res^ detected in the brain homogenates from patients with fCJD^V180I^, a PK-resistant PrP^V180I^ detected in cell lysates also contained diglycosylated form, similar to PrP^res^ in wild-type cell lysates. Furthermore, faint small PK-resistant PrP fragments migrating at ∼17 kDa and ∼7–8 kDa were also detectable in PrP^V180I^ cell lysates. After treatment with PK plus PNGase F, the deglycosylated PrP^res^ was observed in PrP^T183A^, PrP^V180I^, and PrP^Wt^, migrating at ∼20–21 kDa, consistent with our previous observations that 1E4 is able to detect a PrP^Sc^-like form in uninfected brains and cultured cells [10], [11], [18]. A faint band migrating at ∼17 kDa was detectable in all three cell lysates, while a faint ∼7–8 band was mainly found in PrP^V180I^ and PrP^Wt^ (Fig. 5B).

### PrP^V180I^ is present in both cell surface and endoplasmic reticulum

Using immunofluorescence confocal microscope, while PrP^T183A^ was mainly found in the endoplasmic reticulum (ER) (Fig. 5C), evidenced by the co-localization of PrP with calnexin, an ER membrane marker protein, most PrP^Wt^ was observed on the cell surface (Fig. 5E). These results are consistent with previous observations [10], [11]. In contrast, in cells expressing PrP^V180I^ the PrP staining on the cell surface was reduced compared to cells expressing PrP^Wt^ but higher amounts of PrP were colocalized with calnexin in the ER (Fig. 5D).

## Discussion

Five naturally occurring PrP mutations linked to familial prion diseases including D178N, V180I, T183A, F198S, and E200K have reportedly been associated with altered ratios of the three PrP glycoforms. The PrP^T183A^ mutation completely abolishes the first N-glycosylation site, a process that reasonably explains the dramatic decrease or absence of diglycosylated PrP in both PrP^C^ and PrP^Sc^ in the brain and in cultured cells expressing PrP^T183A^ [3], [9]–[11]. In contrast, in patients with the PrP^V180I^ mutation adjacent to N181, it is unclear why the absence of diglycosylated PrP species is evident only in the PK-treated PrP^res^ but not in the untreated PrP in the brain [4].

Because of the close location to N181, the V180I mutation itself was originally suggested as preventing glycosylation at N181 position [19]. However, using the N-linked glycosylation prediction algorithm NetNGlyc 1.0 at http://www.cbs.dtu.dk/services/NetNGlyc/ [11], we predicted a decrease in the glycosylation potential value for N181 in PrP^V180I^ compared to PrP^Wt^ (0.597 vs 0.664) while no potential value was predicted for N181 in PrP^T183A^. The prediction data suggest that although the T183A mutation completely eliminates the N181 glycosylation site, the V180I mutation may merely alter glycan composition at N181, which is consistent with the current experimental data. Moreover, our study with cultured cells revealed that PrP^V180I^ forms PK-resistant diglycosylated PrP^res^ upon PK-treatment, similar to PrP^Wt^ that was observed previously [10], [11]. It is worth noting that ovine PrP^V183I^, equivalent to human PrP^V180I^, also formed PK-resistant diglycosylated PrP in Rov cells when challenged with the scrapie strain 127S [20]. Both *in vivo* and *in vitro* studies consistently indicated that PrP^V180I^ shows typical PrP glycoforms prior to PK-treatment. Furthermore, PrP^V180I^ can form PK-resistant PrP^res^ spontaneously or acquired by exogenous infection, as indicated by *in vitro* cultured cells expressing human PrP^V180I^ or ovine PrP^V183I^. However, these results are inconsistent with the absence of diglycosylated and mono181 PrP^res^ in the brain of fCJD^V180I^.

**Figure 6:**
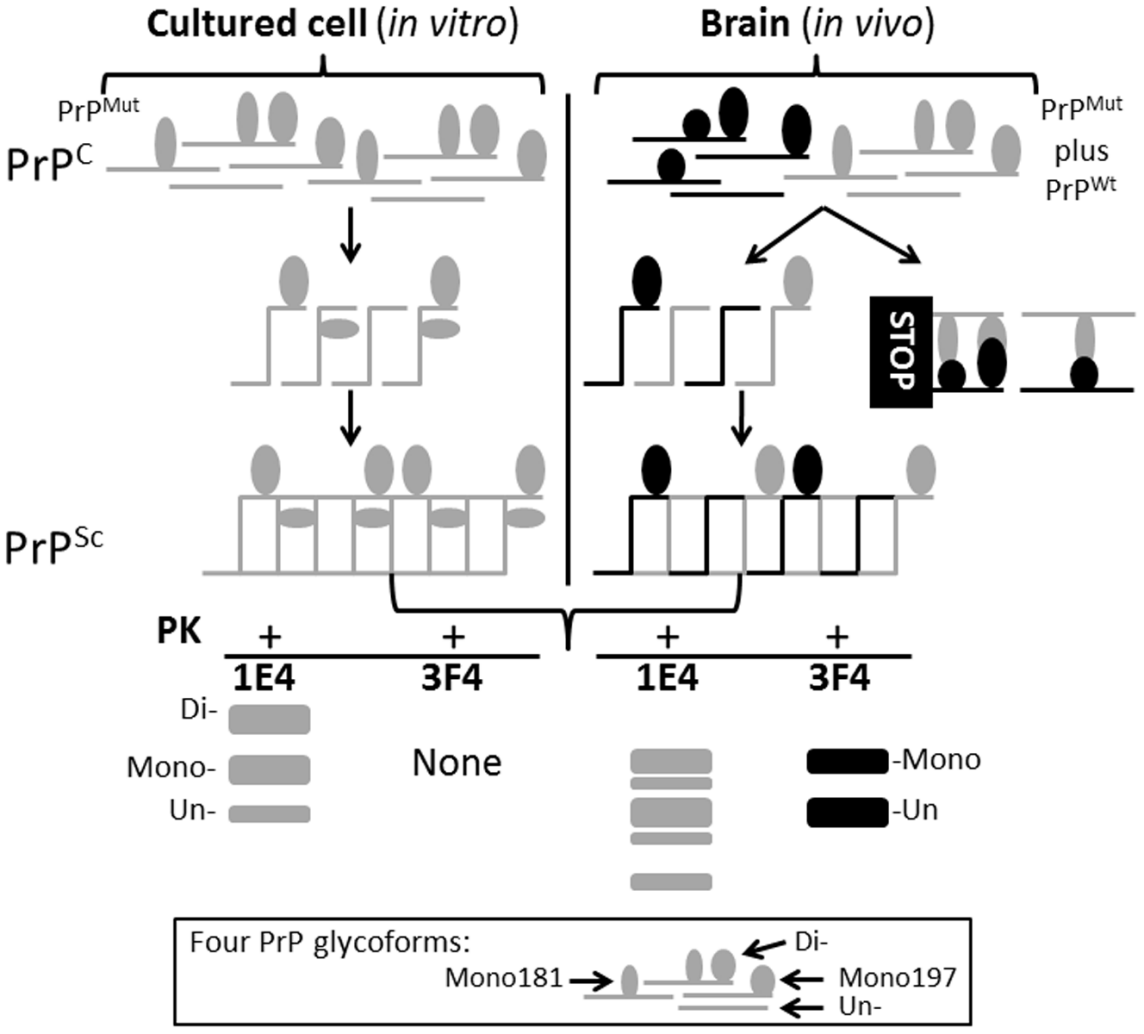
Schematic diagram of glycoform-selective prion formation pathway of PrP^V180I^ in the brain. Four different glycoforms from the PrP^V180I^ mutant allele (gray) are converted into PK-resistant PrP^res^ in cells. However, in the presence of four glycoforms from the wild-type allele (black) in the brain, mono181 and diglycosylated PrP^Wt^ bind to their mutant counterparts, respectively and the PrP^Wt^-PrP^V180I^ complexes are removed from the prion conversion pathway due to possible dominant-negative inhibition. So, only mono197 and unglycosylated PrP are converted into PrP^res^, whereas mono181 and diglycosylated PrP are not. With Western blotting (WB), 1E4 is able to detect three PK-resistant PrP^res^ including the diglycosylated form, while 3F4 does not in cells. In contrast, in the brain tissues, 3F4 detects mono197 and unglycosylated PrP^res^ but not diglycosylated and mono181 PrP^res^. 1E4 detects five PK-resistant PrP^res^ without diglycosylated form.

The discrepancy between the *in vitro* results and the *in vivo* findings may be reconciled as follows. The brains of patients with fCJD^V180I^ contain both mutant and wild-type PrP^V180I^ alleles, whereas cultured cells express only the mutant allele. Therefore, we first hypothesize that the glycoform-selective prion formation pathway observed in the brain involves dominant-negative inhibition caused by the interaction between misfolded and normal PrP molecules. Dominant-negative inhibition has been well documented in a variety of cell and animal models [21]–[26]. Although the mutant alone is convertible in the cultured cells, its conversion is inhibited in the brain. This could be because the interaction of the misfolded PrP caused by the mutation or altered glycans at N181 with its wild-type counterpart may induce a steric hindrance around the PrP N181 region (Fig. 6). As a result, mono197 and unglycosylated PrP^C^ are converted into PrP^Sc^, whereas mono181 and diglycosylated PrP^C^ with the steric hindrance are not (Fig. 6). Our hypothesis may be consistent with the following recent observations. The conformation between the β2 and α2 loop from residues 165 to 175 has been identified as associated with a dominant-negative effect [27], which is also adjacent to the first N-linked glycosylation site. Upon infection of Rov cells with prion 127S, while each of all five mutants that removed the second glycosylation site could form PrP^res^, eight of nine mutants that removed the first glycosylated site could not [20]. Furthermore, in response to ME7 strain challenge, Tg mice lacking the first N-linked site on PrP were resistant, whereas mice lacking the second site were fully susceptible [28]. Interestingly, using the serial protein misfolding cyclic amplification technique, Nishina et al. observed that interactions between different PrP^C^ glycoforms control the efficiency of prion formation involving glycan-associated steric hindrance [29]. Using the same method, the Supattapone group further demonstrated that dominant negative inhibition of prion formation requires no protein X or any other accessory cofactor [30]. Therefore, the region from the loop to the first glycosylation site may be more prone to dominant-negative inhibition by the steric effect. In the case of VPSPr, although there is no PrP mutation, a similar aberrant glycosylation at N181 caused by a rare stochastic event may trigger the processes as described above for fCJD^V180I^. Further investigation on the origin and interaction of alleles and composition of glycans of PrP^Sc^ in the two diseases could address these issues.

Alternatively, the discrepancy between these *in vitro* results and the *in vivo* findings may suggest that the absence of both diglycosylated and mono181 PrP^Sc^ is not attributable to the mutation itself. This possibility is also supported by our present finding that although VPSPr shows no mutations in the PrP open reading frame, PrP^res^ of VPSPr likewise not only lacks the PK-resistant diglycosylated and mono181 species but also shares the same LLEP and the immunoreactivity preference. All these findings suggest that the two diseases bear a similar pathogenetic mechanism and that fCJD^V180I^ is the familial form of VPSPr. It is conceivable that one or more co-factors may be operating in VPSPr and fCJD^V180I^ which may prevent conversion of diglycosylated PrP and mono181 into PrP^Sc^ but trigger conversion of mono197 and unglycosylated PrP to this unique prion strain. There are no reports to date showing that fCJD^V180I^ has been transmitted [31]. Our preliminary study also indicates that the infectivity of PrP^Sc^ from VPSPr seems to be much lower compared to classic sporadic CJD [32], [33]. Although no family history of neurodegenerative disorders has been reported in fCJD^V180I^ cases, eight out of 26 reported VPSPr cases showed a familial history of dementia [7]. Thus, the PrP^Sc^ generated through this peculiar glycoform-selective process is characterized by the presence of a unique conformation that forms LLEP upon PK-treatment and has low infectivity. The other co-factor(s) and how it (they) selectively alters glycosylation at N181 is yet to be determined.

The striking similarity in the physiochemical and biological properties of prions found in fCJD^V180I^ and VPSPr favors the hypothesis that the two conditions share a similar pathogenetic mechanism although one is associated with mutant PrP and the other is not [34]. It is worth noting that because of the long disease duration, multiple PK-resistant PrP fragments, and variable PK-resistance of PrP^Sc^, VPSPr was once suspected to be the sporadic form of GSS associated with PrP^A117V^ mutation (GSS-A117V) [7]. In the same study, nevertheless, we indeed observed different ratios and immunoreactivity of PrP^Sc^ between VPSPr and GSS-A117V [7]. Clearly, whether VPSPr is the sporadic form of fCJD^V180I^ or GSS-A117V remains to be further determined. It is conceivable that cells and animals expressing human PrP^V180I^ or PrP^A117V^ will provide valid models for addressing the outstanding questions mentioned above.

In conclusion, our findings provide the first evidence based on patient samples that glycosylation has a significant role in the formation, selection, and strain trait of prions in spontaneous prion diseases. These findings are significant not only in enhancing our understanding of the molecular mechanism of prion formation but also in developing new therapeutic strategies for prion diseases. In addition, glycoform-selective process present in prion propagation may also occur in other neurodegenerative proteinopathies, notably those sharing prion-like mechanisms [35], [36].

## Materials and Methods

### Subjects and human brain tissues

Nineteen subjects including 10 VPSPr, 6 fCJD^V180I^, 2 sCJD, and one fCJD^T183A^ cases were examined. All were referred to the National Prion Disease Pathology Surveillance Center (NPDPSC, Cleveland, OH) except for an fCJD^V180I^ case from France [4], and two fCJD^V180I^ cases from Japan. The six cases with fCJD^V180I^ were three Caucasian and three Asian patients. Written consent to use autopsy material for research purposes had been obtained from patients or legal guardians for all samples. Clinical data and relevant hospital records were coded and handled according to the protocols approved by the Ethical Committee and Institutional Review Board of Case Western Reserve University to protect patients’ identities. Frozen brain tissues were processed as previously described [7].

### Molecular genetics

The genomic DNA was extracted from frozen brain tissues. The ORF of the *PRNP* was amplified by the polymerase chain reaction (PCR) and PCR products were subjected to automated sequencing and cloned then sequenced to confirm the mutation and polymorphisms as previously described [7].

### Cloning and production of cell lines

M-17 human neuroblastoma cells were transfected with the episomal vector pCEP4β containing the coding sequence of a human wild-type or mutant PrP (T183A or V180I) with valine polymorphism at codon 129 using the cationic lipid DOTAP [Roche Applied Science] and prepared as previously described [9]–[11], [37]. Cell lysates were prepared as described previously [10], [11].

### Preparation of brain homogenate, S2, and P2 fractions

The 10% (w/v) brain homogenates were prepared in 9 volumes of lysis buffer (10 mM Tris, 150 mM NaCl, 0.5% Nonidet P-40, 0.5% deoxycholate, 5 mM EDTA, pH 7.4) with pestle on ice. When required, brain homogenates were centrifuged at 1,000 g for 10 min at 4°C. In order to prepare S2 and P2 fractions, the supernatants (S1) were further centrifuged at 35,000 rpm (100,000 g) for 1 hour at 4°C. After the ultracentrifugation, the detergent-soluble fraction was recovered in the supernatants (S2) while the detergent-insoluble fraction (P2) was recovered in the pellets that were resuspended in lysis buffer as described [18].

### Specific capture of PrP^Sc^ by gene 5 protein and immunoprecipitation of PrP by 6H4 antibody

The preparation of gene 5 protein (g5p) conjugated magnetic beads and specific capture of PrP^Sc^ by g5p beads were conducted as previously described [18]. The immunoprecipitation of PrP from brain homogenates and cell lysates by 6H4-conjugated magnetic beads was performed as previously described [16], [18].

### Immunoblotting

Samples treated with or without PK-digestion were resolved on 15% Tris-HCl Criterion (Bio-Rad) as described previously [18]. The following anti-PrP antibodies were used (Fig. S1): mouse monoclonal antibody (mAb) 3F4 directed against human PrP residues 106–110 [38], [39], 1E4 against human PrP residues 97–105 [10], [18], 6H4 against human PrP144–152 (Prionics AG, Switzerland), V14 recognizing the human PrP185–196 [11]-[13], Pc248 directed against the octarepeat region [12], [40], and Bar209 anti-mono197 and unglycosylated PrP mAb [12] (Fig. S1), recognizing a conformational epitope [12] likely involving human PrP168-181, like anti-mono197 PrP mAb V61 [12].

### Immunofluorescence and confocal microscopy

Immunofluorescence staining of PrP in transfected cells expressing PrP^Wt^, PrP^V180I^, or PrP^T183A^ was performed as previously described [11]. In brief, the cells were fixed in 4% paraformaldehyde. After blocking with PBST (10% Goat Serum, 2% T-20, 1% Triton X-100), the cells were then incubated with 3F4 (1∶10,000) or calnexin (1∶1,000) at room temperature, rinsed with PBS, followed with FITC-conjugated goat anti-mouse IgG at 1∶1,000 (Sigma, St. Louis, MO) or Alexa Fluor 568-conjugated goat anti-rabbit as secondary antibody. Microscopy was performed using a high-speed Leica SP5 Broadband confocal microscope (Wetzlar, Germany) and HCX PL APO CS 63× oil immersion objective (NA 1.4). Images were acquired and analyzed using Leica Application Suite software. Further analysis for colocalization and correlation was performed using Imaris imaging suite (Bitplane, St. Paul, MN).

## Supporting Information

Figure S1 **Schematic diagram of the NMR-derived structure of human PrP (1) and the epitopes of anti-PrP antibodies used in this study.** The five black five-point stars represent the octapeptide repeats between residues 51 and 91. The two black right arrows represent the β-sheets. The three black waves represent the α-helical structures. The two black 7-point stars represent the two N-linked glycans at residues 181 and 197. The known epitopes of the five antibodies are indicated including Pc248, 1E4, 3F4, 6H4, and V14. Bar209 has a conformational epitope (12), which likely involves PrP168–181 as it recognizes PrP^C^ depending on N181 occupancy, like V61 mAb (12).
(TIF)

Figure S2 **Characterization of the Bar209 antibody by Western blotting with specific PrP glycoforms.** The following brain homogenates containing different PrP glycoforms were used (13): tga20 mouse expressing wild type mouse PrP containing largest amount of di-, intermediate mono-, and smallest amount of un-glycosylated PrP species (lane 1); Tg mouse expressing mono197 and unglycosylated PrP without mono181 because of the mutation at the first glycosylation site (arrowhead) (lane 2); Tg mouse expressing mono181 and unglycosylated PrP without mono197 because of the mutation at the second glycosylation site (arrow) (lane 3); and Tg mouse expressing unglycosylated PrP only because of the mutations at both glycosylation sites (lane 4). While the control Pc248 antibody (directed against the anti-octarepeat region of PrP^C^) is able to detect all four PrP glycoforms including di-, mono197, mono181, and un-glycosylated PrP, Bar209 only detects mono197 and unglycosylated PrP species.
(TIF)

Figure S3 **Reactivity of RCA**–**I with PrP glycans.** PrP was immunoprecipitated by 6H4 from brain homogenates of sCJD, VPSPr, and fCJD^V180I^ and probed with RCA–I. As a control, the brain homogenate from sCJD directly loaded onto the gel was probed with 3F4.
(TIF)
