# Correction: Glycoform-Selective Prion Formation in Sporadic and Familial Forms of Prion Disease

**DOI:** 10.1371/annotation/5391f30a-0875-4145-a1ea-74aedbbcd1e4

**Published:** 2013-10-09

**Authors:** Xiangzhu Xiao, Jue Yuan, Stéphane Haïk, Ignazio Cali, Yian Zhan, Mohammed Moudjou, Baiya Li, Jean-Louis Laplanche, Hubert Laude, Jan Langeveld, Pierluigi Gambetti, Tetsuyuki Kitamoto, Qingzhong Kong, Jean-Philippe Brandel, Brian A. Cobb, Robert B. Petersen, Wen-Quan Zou

There are multiple in-text citation errors in the Methods section and Figure Legends. The second sentence in the Methods section under the subheading “Immunoblotting” should end with: “...and Bar209 anti-mono197 and unglycosylated PrP mAb [14] (Fig. S1 and S2), recognizing a conformational epitope [14] likely involving human PrP168-181, like anti-mono197 PrP mAb V61 [12].”

The last sentence of the Figure S1 Legend should be: “Bar209 has a conformational epitope (14), which likely involves PrP168–181 as it recognizes PrPC depending on N181 occupancy, like V61 mAb (12).”

The first sentence of the Figure S2 Legend should begin: “The following brain homogenates containing different PrP glycoforms were used [21]...” 

