# Supplementary figures and images for: Glycoform-Selective Prion Formation in Sporadic and Familial Forms of Prion Disease
Source: PLoS One. 2013 Mar 19;8(3):e58786. doi: 10.1371/journal.pone.0058786 (PMC3602448; doi:10.1371/journal.pone.0058786)

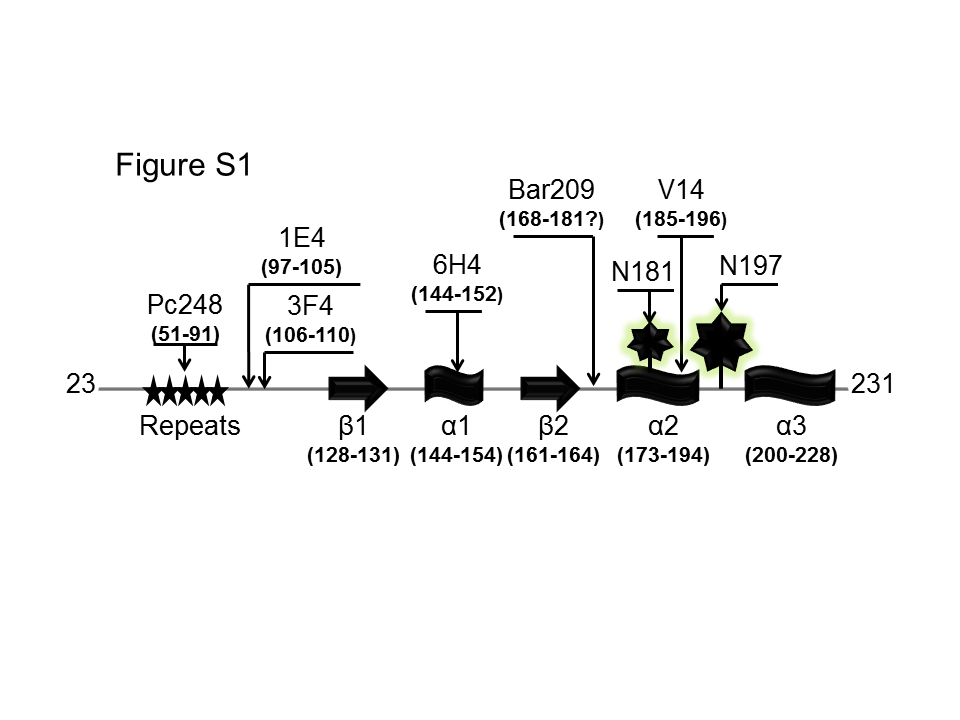

Supplement: Figure S1 — Schematic diagram of the NMR-derived structure of human PrP (1) and the epitopes of anti-PrP antibodies used in this study. The five black five-point stars represent the octapeptide repeats between residues 51 and 91. The two black right arrows represent the β-sheets. The three black waves represent the α-helical structures. The two black 7-point stars represent the two N-linked glycans at residues 181 and 197. The known epitopes of the five antibodies are indicated including Pc248, 1E4, 3F4, 6H4, and V14. Bar209 has a conformational epitope (12), which likely involves PrP168–181 as it recognizes PrPC depending on N181 occupancy, like V61 mAb (12). (TIF) [file pone.0058786.s001.tif]

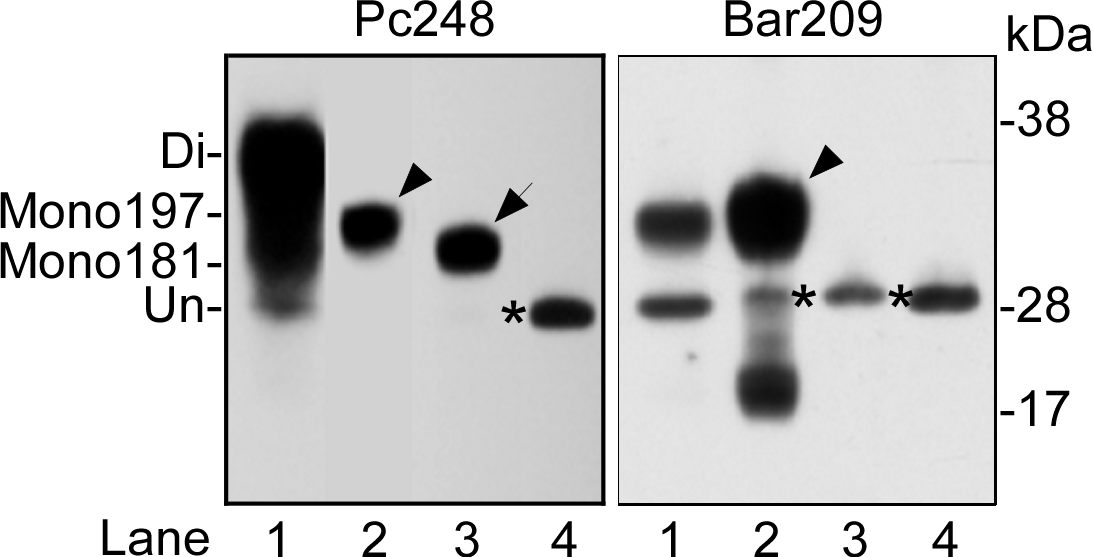

Supplement: Figure S2 — Characterization of the Bar209 antibody by Western blotting with specific PrP glycoforms. The following brain homogenates containing different PrP glycoforms were used (13): tga20 mouse expressing wild type mouse PrP containing largest amount of di-, intermediate mono-, and smallest amount of un-glycosylated PrP species (lane 1); Tg mouse expressing mono197 and unglycosylated PrP without mono181 because of the mutation at the first glycosylation site (arrowhead) (lane 2); Tg mouse expressing mono181 and unglycosylated PrP without mono197 because of the mutation at the second glycosylation site (arrow) (lane 3); and Tg mouse expressing unglycosylated PrP only because of the mutations at both glycosylation sites (lane 4). While the control Pc248 antibody (directed against the anti-octarepeat region of PrPC) is able to detect all four PrP glycoforms including di-, mono197, mono181, and un-glycosylated PrP, Bar209 only detects mono197 and unglycosylated PrP species. (TIF) [file pone.0058786.s002.tif]

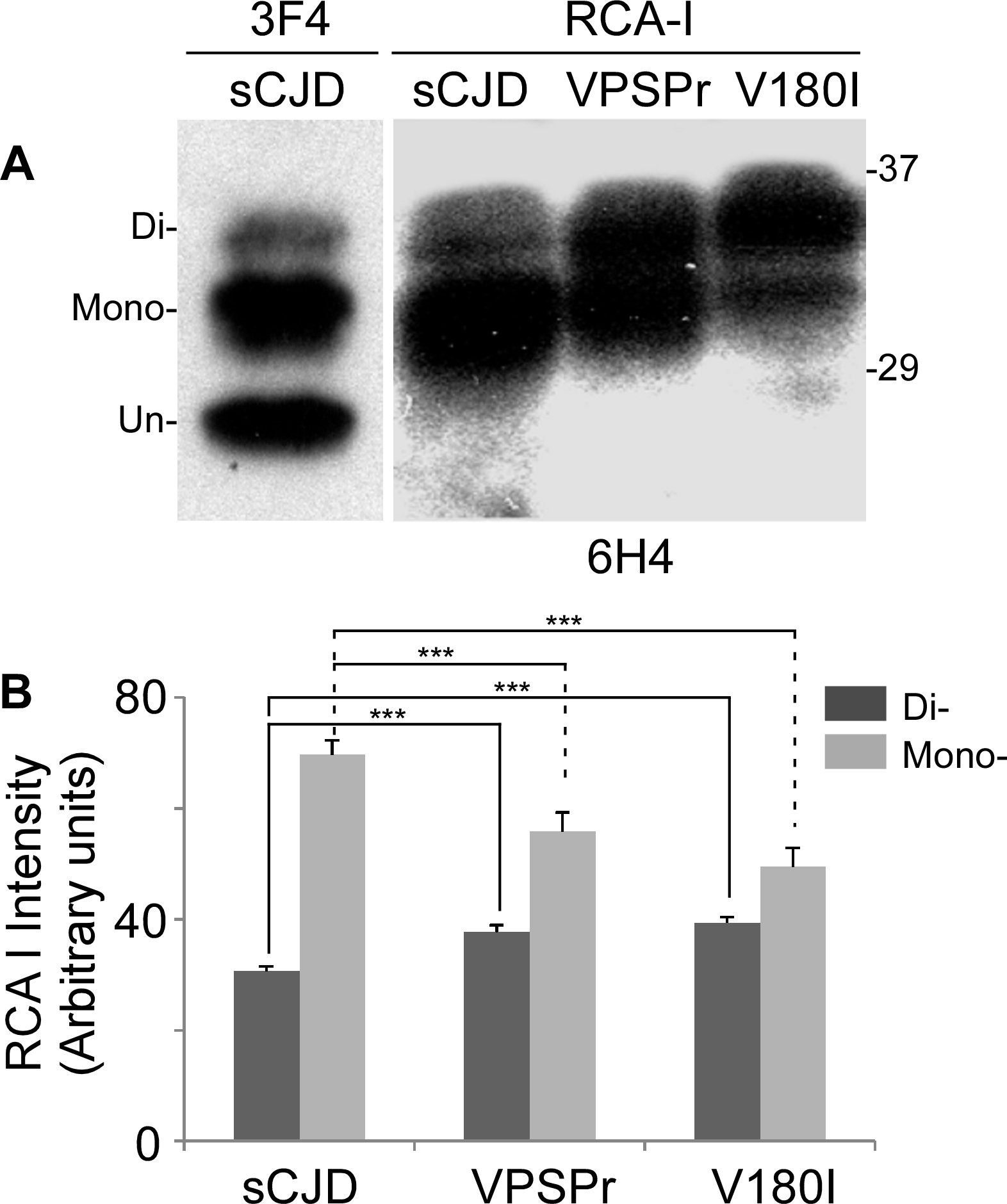

Supplement: Figure S3 — Reactivity of RCA–I with PrP glycans. PrP was immunoprecipitated by 6H4 from brain homogenates of sCJD, VPSPr, and fCJDV180I and probed with RCA–I. As a control, the brain homogenate from sCJD directly loaded onto the gel was probed with 3F4. (TIF) [file pone.0058786.s003.tif]
